# Plasma MicroRNA-16 Is a Biomarker for Diagnosis, Stratification, and Prognosis of Hyperacute Cerebral Infarction

**DOI:** 10.1371/journal.pone.0166688

**Published:** 2016-11-15

**Authors:** Chunou Tian, Zifu Li, Zhigang Yang, Qinghai Huang, Jianmin Liu, Bo Hong

**Affiliations:** 1 Department of Neurosurgery, Changhai Hospital, Second Military Medical University, Shanghai, China; 2 Department of Neurosurgery, Number 422 Hospital of PLA, Zhanjiang, Guangdong, China; Universita degli Studi di Napoli Federico II, ITALY

## Abstract

Indices for the diagnosis of hyperacute cerebral infarction (HACI) and the prediction of prognosis are essential for timely and appropriate management. MicroRNAs (miRNAs) that regulate gene expression following stroke have potential use as prognostic markers of HACI. Here, we explored whether concentrations of circulating miRNAs correlate with clinical outcomes and thus form a system of stroke stratification. Plasma samples from patients with HACI (n = 7) and age-matched healthy volunteers (HVT, n = 4) were screened by microarray to find differentially expressed miRNAs, some of which were further verified by quantitative reverse transcription polymerase chain reaction (qRT-PCR) (HACI:HVT = 33:23). The target genes of the miRNAs with verified differential expression were investigated by GO and KEEG analyses. Using the TOAST (OCSP) criteria and the 3-month modified Rankin Score (mRS), relationships among the expression patterns of specific miRNAs, stroke stratification, and clinical prognosis were determined. The microarray analysis revealed 12 differentially expressed miRNAs. Among seven selected miRNAs verified with qRT-PCR, miR-16 expression in the HACI group was the most significantly different from the HVT group (P < 0.01). Bioinformatics analysis showed that the potential target genes of miR-16 were mainly involved in programmed cell death and the p53 signaling pathways. Receiver operating characteristic (ROC) analysis showed that the area under the curve (AUC) of miR-16 was 0.775 (sensitivity 69.7% and specificity 87%) and 0.952 (sensitivity 100% and specificity 91.3%) in overall patients and patients with large artery atherosclerosis (LAAS), respectively. Elevated miR-16 expression was associated with the stroke subtype of LAAS, total anterior circulation infarction, partial anterior circulation infarction, and poor prognosis (P < 0.05). A diagnostic method based on rapid measurement of plasma miR-16 has the potential to identify hyperacute cerebral infarction with LAAS with high sensitivity and specificity, which would inform and improve early treatment decisions and disease management.

## Introduction

Worldwide, stroke severely reduces the quality of life of its victims, and has recently risen from the second to the first leading cause of death in China [1]. Ischemic stroke accounted for 70–85% of strokes.

The effectiveness of treatments for hyperacute cerebral infarction (HACI), including intravenous thrombolysis or thrombectomy, is usually confined to within 6 hours of the ischemic event [2]. Therefore, rapid diagnosis is pivotal for clinical decisions, even before the infarction lesions can be observed by neuroimaging.

Robust attempts to identify circulating protein markers or small molecule biomarkers for stroke have been performed in animal models or with metabolomics analysis of clinical samples [3–6]. MicroRNAs (miRNAs), varying in length from 18–25 nucleotides, are small, non-coding, endogenous RNA molecules. Numerous miRNAs have been implicated in the pathogenesis of stroke by evidence showing them regulating gene expression at multiple epigenetic levels [7–8]. A number of studies have sought to identify differentially regulated circulating miRNAs in stroke patients and validate them as biomarkers [9–11]. However, the existence of multiple stroke etiologies and classifications has complicated the challenge of finding diagnostic markers at the hyperacute stage that have high sensitivity and specificity.

The present study aimed to identify specific circulating miRNAs that would facilitate the diagnosis of hyperacute cerebral infarction (<6 hours after stroke) and validate their usefulness in accurate prognosis. The miRNAs microarray data and clinical stratification with criteria from the Trial of org10172 in Acute Stroke Treatment (TOAST) and the Oxford Community Stroke Project (OCSP) [12] were used to test correlations between circulating levels of selected miRNAs and stroke subtypes together with their prognoses.

## Materials and Methods

### Patients

The study protocol was approved by the Medical Ethics Committee of Shanghai Changhai Hospital and carried out according to the principles of the Declaration of Helsinki. We designed this study in March of 2014. Consecutive patients with HACI admitted to the Cerebrovascular Diseases Center in Shanghai Changhai Hospital between July 2014 and January 2016 were enrolled in the study after written informed consent was obtained, following the inclusion and exclusion criteria. The date range in which human subjects’ samples were collected was eighteen months. Inclusion criteria: time duration from stroke onset to admission was less than 6 hours. Exclusion criteria: immune disease, trauma, coronary heart disease, organ failure, tumor, and infection. The control population of healthy volunteers (HVT) was made up of healthy people matched by gender and age to the study group.

### Plasma collection and RNA preparation

Blood samples from the HACI group were obtained in the emergency room, and immediately centrifuged. The supernatants were transferred to RNase-free tubes and stored at -80°C. Relevant clinical data were provided through clinical or outpatient follow-up. Total RNA was extracted from 100 μl plasma according to the instructions of the S/P RNAiso kit (Shenzhen Ongran Biotech, Shenzhen, China) containing *Caenorhabditis elegans* miRNA (cel-miR-54) as a spiked-in control. At the time of sample collection we recorded information that could identify individual participants via the medical records system of Changhai Hospital.

### MiRNAs microarray hybridization

Human miRNAs microarrays (Agilent Human miRNA (8*60K) V19.0 array) from Agilent Technologies were used. The database (miRBase 19.0) contained 2006 human miRNAs. MiRNAs samples were labeled by the Complete Labeling and Hyb Kit (Agilent Technologies, Santa Clara, CA, US), and hybridized with 100 ng Cy3-labeled RNA. The microarray image information was converted into spot intensity values using Feature Extraction Software 10.7. Raw data were normalized by the Quantile algorithm (Gene Spring Software 12.6). Any sample that showed intra-array coefficients of variation (CV) above 15% was excluded from further analysis.

### Quantitative real-time PCR

MiRNAs were quantified by using an S-Poly(T) miRNA qPCR-assay (Shenzhen Ongran Biotech, Shenzhen, China) according to the protocol of the manufacturer [13]. The Ct was defined as the fractional cycle number at which the fluorescence exceeded the given threshold. Ct values from qPCR assays greater than 35 were rejected. The plasma levels of miRNAs were detected and analyzed by investigators who were blinded to the clinical data. Relative expression was normalized to the spiked-in control in triplicate and was calculated by the comparative Ct method (2^-ΔΔCt^).

### Bioinformatics analysis

The target genes of miR-16 were predicted via miRwalk suite (http://www.umm.uni-heidelberg.de/apps/zmf/mirwalk/mirnatargetpub.html) [14]. Algorithms containing miRanda, TargetScan, and RNAhybrid were used. The combination of the three tools provided a robust approach to identifying highly predictive, potentially functionally relevant miRNA-target gene interactions. The criteria of our search was a minimum miRNA seed length of 7 nucleotides and the presence of binding sites on the 3'-UTR of the target mRNA. Targets predicted by at least two algorithms of the three with P ≤ 0.05 were considered as candidate targets. Targets identified by miRwalk that were also experimentally validated in published reports were selected. To explore the functions of miR-16, we included experimentally validated targets and performed gene ontology (GO) analysis to display the 20 GO categories with the most significant differences. The significantly affected signaling pathways were predicted based on the Kyoto Encyclopedia of Genes and Genomes (KEGG) and BioCarta with a P value threshold of 0.01. GO and KEGG analyses were performed using DAVID tools (http://david.abcc.ncifcrf.gov/).

### Patient follow-up strategy

Follow-up was carried out 3 months after the onset of cerebral infarction to record clinical outcomes. Outpatient and telephone follow-ups were used. A good outcome was defined as mRS 0–2 and poor as mRS 3–6.

### Correlation analysis of miRNAs with stroke

Univariate and multivariate logistic regressions were performed to observe the correlations between candidate miRNA biomarkers and HACI status. ROC curves were used to evaluate the diagnostic accuracy of selected miRNAs.

### Correlation between specific miRNAs and stroke stratification/prognosis

HACI patients were classified according to TOAST and OCSP criteria. The expression levels of miRNA biomarkers between HACI patients in different subtypes and HVT were compared. The differences between the good and poor prognosis groups were also examined.

### Statistical analysis

The qualitative data were compared with a χ^2^ test. When the quantitative data fit the normal distribution (the Shapiro-Wilk test, α = 0.1) and the homogeneity of variance (Levene’s test), a t-test or one-way analysis of variance (ANOVA) was applied. For the quantitative data that did not fit the normal distribution or the homogeneity of variance, the Mann-Whitney test or the Kruskal-Wallis test was performed. Univariate and multivariate logistic regression were performed with the “Forward:Wald” method (Entry 0.1, Removal 0.15). All statistical calculations were performed using the Statistical Program for Social Sciences (SPSS) software 21.0 (IBM, Armonk, New York, US).

## Results

### Patients for microarray analysis and qPCR validation

Forty patients with HACI and thirty HVT were recruited in this study. Seven HACI and four HVT were selected randomly (simple random sampling, random number table) for microarray analysis. Thirty-three HACI and 26 HVT were selected for qPCR validation. After excluding samples whose cycle threshold (Ct) was more than 35, 33 HACI and 23 HVT were used for qPCR data processing.

### Patient stratification with TOAST and OCSP criteria

According to TOAST criteria, the seven HACI patients in the microarray group were classified as LAAS (n = 1), cardio-embolic infarct (CEI, n = 4), or undetermined etiology (UDE, n = 2). According to OCSP criteria, they were classified as TACI (n = 4), PACI (n = 2), or posterior circulation infarct (POCI, n = 1).

According to TOAST criteria, 33 HACI patients in the qPCR group were classified by etiologic diagnosis as LAAS (n = 9), CEI (n = 10), small artery occlusion (SAO, n = 8), other determined etiologies (ODE, n = 1), or UDE (n = 5). The miRNA expression patterns of HACI patients in different TOAST subtypes and HVT were compared.

According to OCSP criteria, 33 HACI patients in the qPCR group were classified as TACI (n = 4), PACI (n = 17), POCI (n = 7), or lacunar cerebral infarct (LACI, n = 5). We compared the miRNA expression levels of HACI patients in different OCSP subtypes and HVT.

### Microarray analysis of differences in miRNA expression between HACI and HVT groups

**Fig 1:**
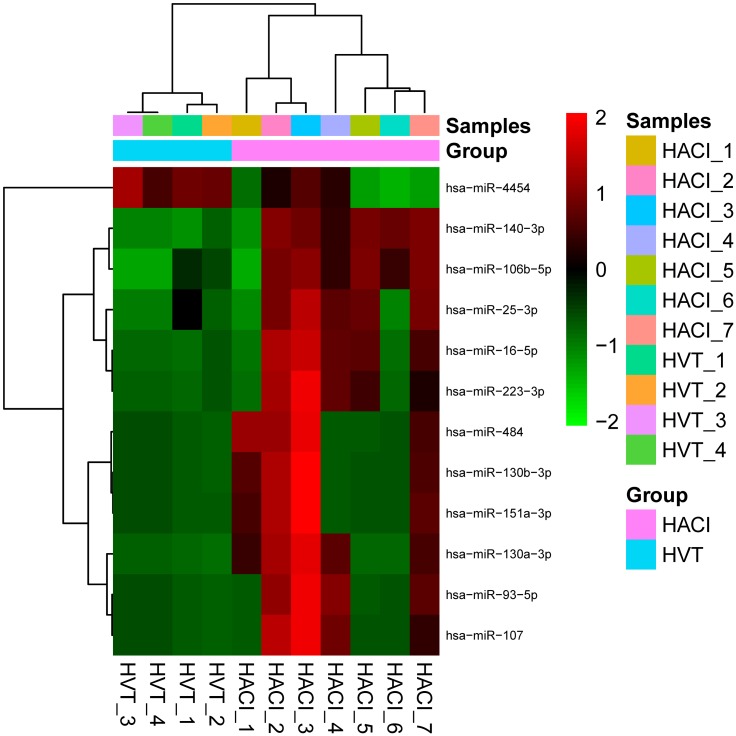
Clustering heat map of microarray data showing differential expression of miRNAs. A t-test was performed for the expression levels of each miRNA comparing the group of seven HACI to the group of four HVT. The twelve miRNAs with the most significant differences (fold change >2, P < 0.05) are presented. The heat map was drawn using normalized data from the microarray experiment. The logged values of the normalized data were adjusted by subtracting the median and then subjected to hierarchical clustering using R Software. The color scale shown on the right side illustrates the relative expression level of a miRNA across all samples: red color represents an expression level above the mean, green color represents expression lower than the mean. Individual samples of plasma were ranked and grouped according to the distribution of their miRNA signals in the clustering heat map. Four cases of HVT in our study were linked into one group.

**Fig 2:**
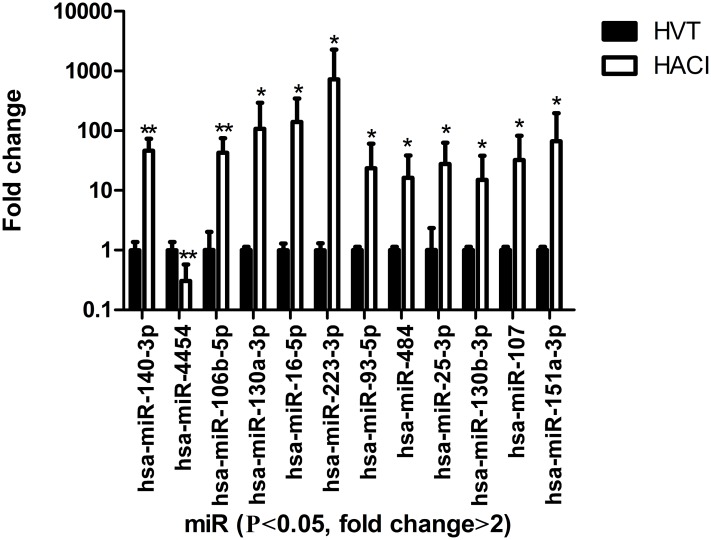
Microarray data showing the different expression profiles of HACI and HVT patients. Each fold change was calculated from the base 2 power of the normalized data of the miRNA microarray. The fold changes equaled the ratio between the mean of the values of the HACI group and the mean of the values of the HVT group. The heights of the columns in the chart represent the fold change; the bars represent one standard deviation. *P < 0.05 and **P < 0.01.

Microarray technology was used to detect the expression levels of plasma miRNAs. Expression differences were compared among seven HACI patients and four HVT. MiRNAs with a change in expression that was more than two-fold and having a P value less than 0.05 were identified. Then a clustering heat map (Fig 1) was used to visualize 11 miRNAs that were upregulated (miR-140, miR-106b, miR-130a, miR-16, miR-223, miR-93, miR-484, miR-25, miR-130b, miR-107, and miR-151) and one miRNA that was down-regulated (miR-4454) (Fig 2). MiR-16 and miR-223 were specific to cerebral tissue [15].

### Validation of differential expression by qPCR

**Fig 3:**
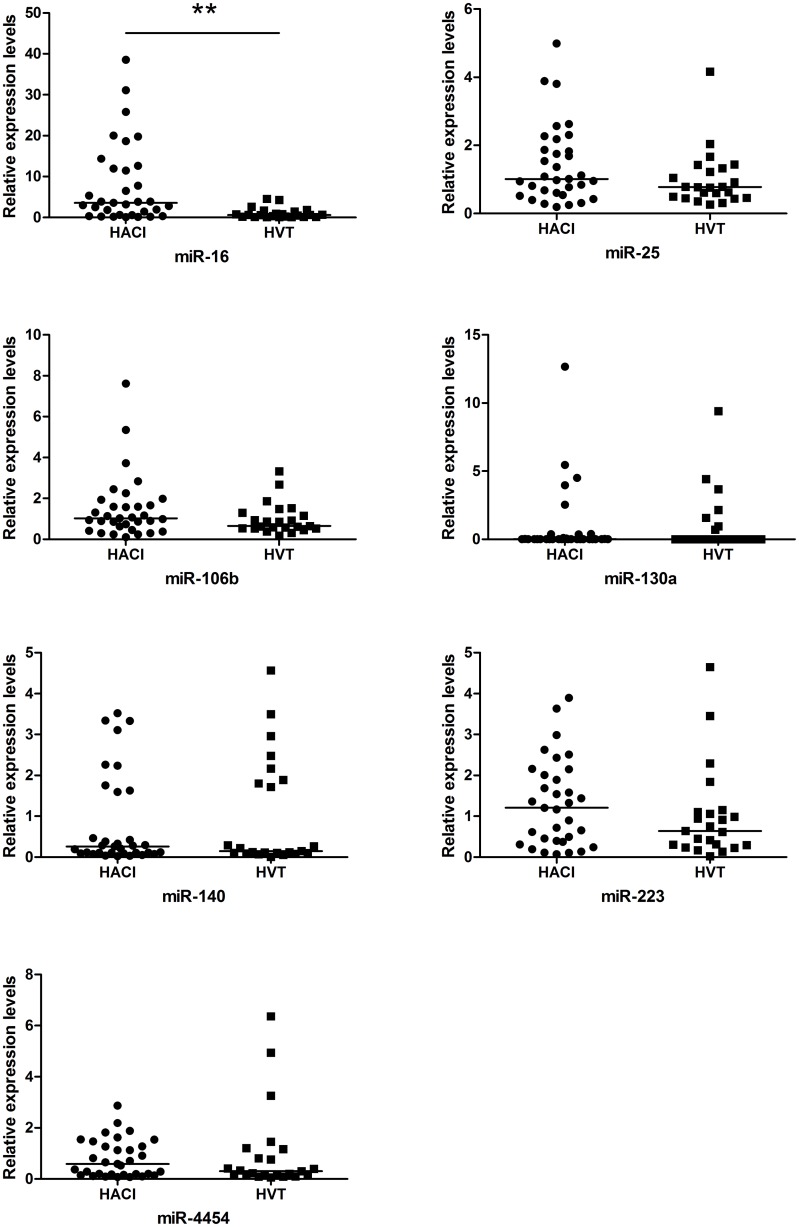
Relative expression levels of miRNAs measured by qPCR, comparing the HACI and HVT groups. The straight line in the figure represents the median. The original Ct value was treated with the ΔΔCt method. *P < 0.05 and **P < 0.01.

A miRNA was chosen for qPCR validation from the different expression profiles of the microarray if the mean of the normalized values was greater than 1.0 in at least one group (HACI or HVT). Seven of 12 miRNAs identified by the microarray as being differentially expressed betweenthe HACI and HVT group were selected for validation. The median, calculated by Ct values, of the seven miRNAs in the HACI group were all higher than the corresponding medians in the HVT group, but the only miRNA that showed a statistically significant difference between the two groups was miR-16 (P < 0.01). There were no significant differences between the two groups for miR-25, miR-106b, miR-130a, miR-140, miR-223, and miR-4454 (Fig 3).

### Bioinformatics analysis of miRNA as a biomarker

**Fig 4:**
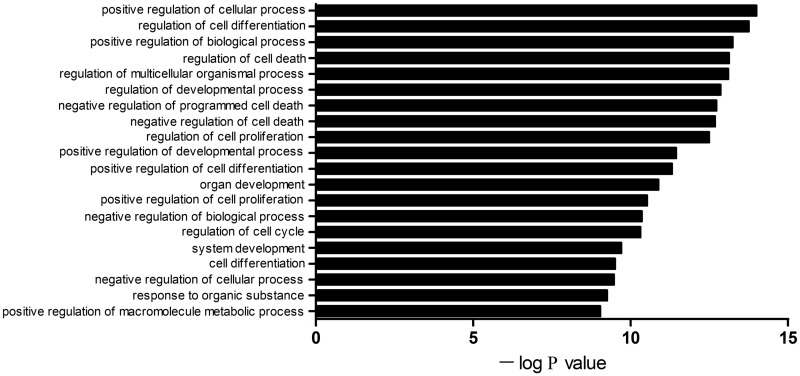
GO categories for putative target genes of miR-16. The abscissa represents the negative logarithm (base 10) of the P value which comes from enrichment analysis. The 20 GO categories (biological processes) with the smallest P value are displayed.

**Fig 5:**
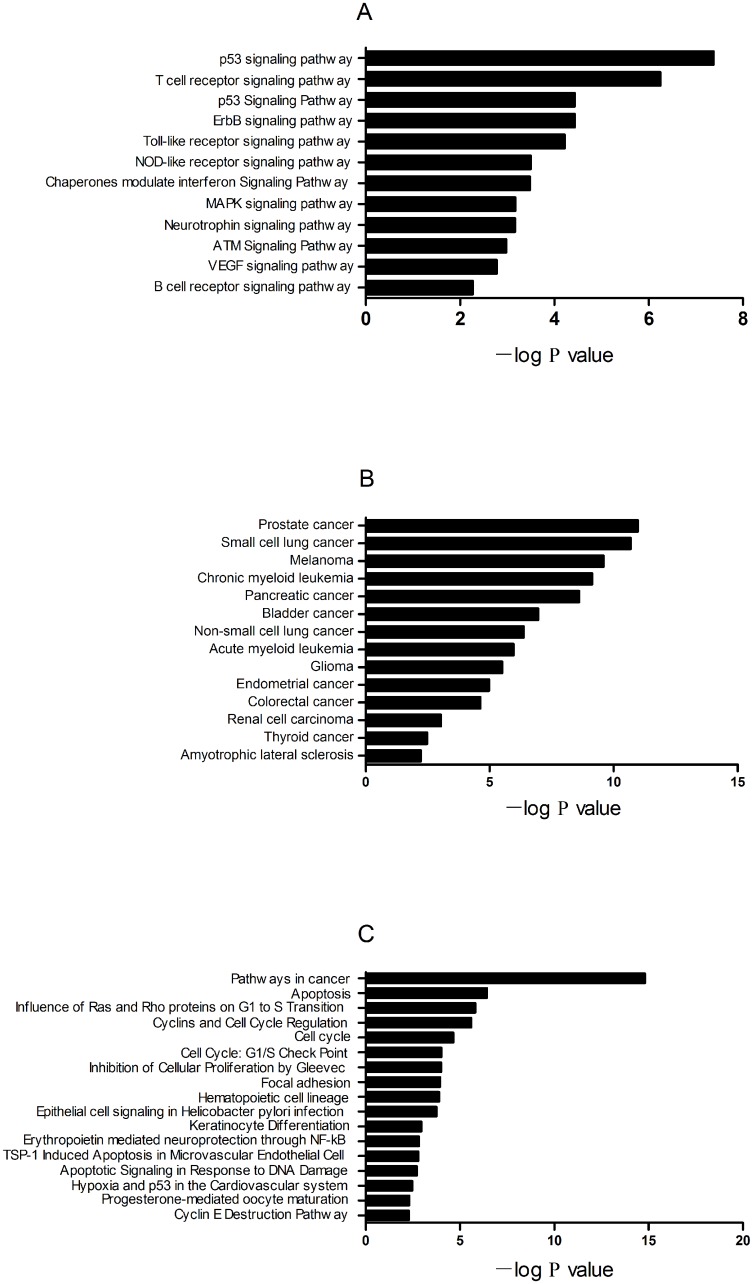
Pathway analysis based on putative target genes of miR-16. Signaling pathways were predicted based on analysis with KEGG and BioCarta using a P value threshold of 0.01. (A)Classical signaling pathways. (B) Signaling pathways related todiseases. (C) Other signaling pathways.

A total of 1495 genes were predicted to be targets of miR-16 during hyperacute cerebral infarction. Fig 4 shows the 20 GO categories with the most significant differences. The gene ontology categories covered three domains: biological processes, cellular components, and molecular functions. The domain of biological processes have been presented in Fig 4. Enrichment analysis was used to obtain a P value for every GO and KEGG category. The GO categories with the smallest P values were cell differentiation, cell death, developmental processes, programmed cell death, cell proliferation, and cell cycle (Fig 4). The 43 enrichment pathways with the smallest P value could be roughly divided into three categories: classical signaling pathways, disease, and other. Predicted target genes related to the following classical signaling pathways: p53, T cell receptors, ErbB, Toll-like receptors (TLR), NOD-like receptors, and MAPK (Fig 5).

### Regression model for miR-16

Table 1 shows the clinical features of the HACI and HVT groups. Most clinical features showed no significant difference between the two groups. Exceptions were atrial fibrillation, diastolic blood pressure, triglycerides, and white blood cells (P < 0.05). Univariate logistic regression with miR-16 showed an odds ratio (OR) of 1.621 (95% confidence interval (CI) 1.095–2.399, P = 0.016) (Table 2). We performed multivariate logistic regression analysis with atrial fibrillation, diastolic blood pressure, triglycerides, white blood cells, and miR-16. Lastly, only atrial fibrillation [OR 9.268, 95% CI 0.854–100.637, P = 0.067, x_1_] and miR-16 (OR 1.669, 95% CI 1.071–2.602, P = 0.024, x_2_) were entered into the regression model. The regression model was as follow: Y = -1.123 + 2.227x_1_ + 0.513x_2_ (Table 3). Atrial fibrillation had no statistical significance in the model.

**Table 1:** Clinical features of the HACI group and the HVT group.

Features	HACI (n = 33)	HVT (n = 23)	P
Age (years)	68 (13)	63.70±14.31	0.474
Male/female (n/n)	23/10	17/6	0.731
Current smoking, n (%)	9 (27.2)	9 (39.1)	0.35
DM, n (%)	8 (24.2)	3 (13.0)	0.299
Hypertension, n (%)	22 (66.7)	17 (73.9)	0.562
Hyperlipidemia, n (%)	14 (42.4)	15 (65.2)	0.093
CHD, n (%)	4 (12.1)	2 (8.7)	1
Alcohol intake, n (%)	2 (6.1)	2 (8.7)	1
Atrial fibrillation, n (%)	10 (30.3)	1 (4.3)	0.039
SBP (mmHg)	144.18±18.43	135 (22)	0.629
DBP (mmHg)	82 (12.5)	79 (14)	0.043
Body temperature (°C)	36.2 (0.55)	36.2 (1.5)	0.931
Heart rate (min^-1^)	80 (14)	76 (10)	0.236
Blood-glucose (mmol/L)	6.1 (3.2)	6 (1)	0.874
TC (mmol/L)	5.04±1.21	4.72±0.99	0.305
TG (mmol/L)	1.20 (0.67)	1.58 (1.14)	0.020
LDL (mmol/L)	2.97 (0.94)	2.96±0.72	0.918
HDL (mmol/L)	1.26±0.34	1.19 (0.59)	0.627
Cr (μmol/L)	72 (34	67.48±13.63	0.197
WBC (10^9^/L)	8.25 (3.18)	7.01±1.81	0.020
HGB (g/L)	138.22±19.68	143.57±13.92	0.269
PLT (10^9^/L)	187 (71.25)	209.17±60.16	0.394
RBC (10^12^/L)	4.58±0.67	4.67±0.43	0.536
PT (s)	13.67±0.80	13.3 (0.95)	0.152
APTT (s)	36.82±4.34	35.5±4.89	0.344
FIB (g/L)	3.16±0.75	3.00±0.93	0.504
D-dimer (μg/mL)	1.01 (3.74)	0.265 (3.30)	0.102
FDP (μg/mL)	6.01 (8.92)	0.78 (15.95)	0.253
MiR-16	3.57 (11.72)	0.6 (1.35)	0.001

DM, diabetes mellitus; CHD, coronary heart disease; SBP, systolic blood pressure; DBP, diastolic blood pressure; TC, total cholesterol; TG, triglyceride; LDL, low-density lipoprotein; HDL, high-density lipoprotein; Cr, creatinine; WBC, white blood cell; HGB, hemoglobin; PLT, platelet; RBC, red blood cell; PT, prothrombin time; APTT, activated partial thromboplastin time; FIB, fibrinogen; FDP, fibrin degradation product.

**Table 2:** Univariate logistic regression.

Item	B	S.E.	Wald	df	P	OR	95% CI of OR	
							Lower limit	Upper limit
MiR-16	0.483	0.200	5.826	1	0.016	1.621	1.095	2.399
constant	-0.749	0.423	3.128	1	0.077	0.473		

**Table 3:** Multivariate logistic regression.

Item	B	S.E.	Wald	df	p	OR	95% CI of OR	
							Lower limit	Upper limit
MiR-16	0.513	0.226	5.123	1	0.024	1.669	1.071	2.602
Atrial fibrillation	2.227	1.217	3.348	1	0.067	9.268	0.854	100.637
constant	-1.123	0.492	5.211	1	0.022	0.325		

### Specificity and sensitivity of miR-16 for HACI stroke patients

**Fig 6:**
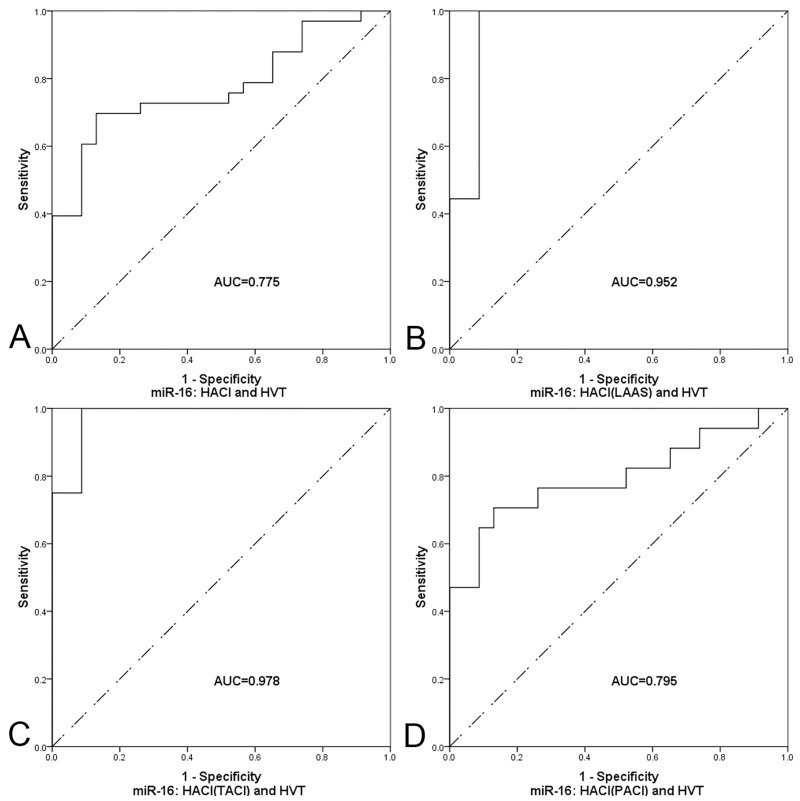
Evaluation of plasma miR-16 for the diagnosis of HACI. ROC curves comparing plasma miR-16 expression levels between the HACI group and other group(s). (A) HACI group and HVT group. (B) HACI group with LAAS and HVT groups. (C) HACI group with TACI and HVT groups. (D) HACI group with PACI and HVT groups. The diagonal in each figure is for reference.

In order to study the diagnostic accuracy of miR-16 as a biomarker for HACI, a ROC curve was drawn (Fig 6A). The area under the curve (AUC) was 0.775 (95% CI 0.653–0.897, P = 0.001). When the relative expression level of miR-16 was at the optimum truncation point (1.8333), sensitivity and specificity were 69.7% and 87%, respectively.

### Correlation between miR-16 and stroke stratification

**Fig 7:**
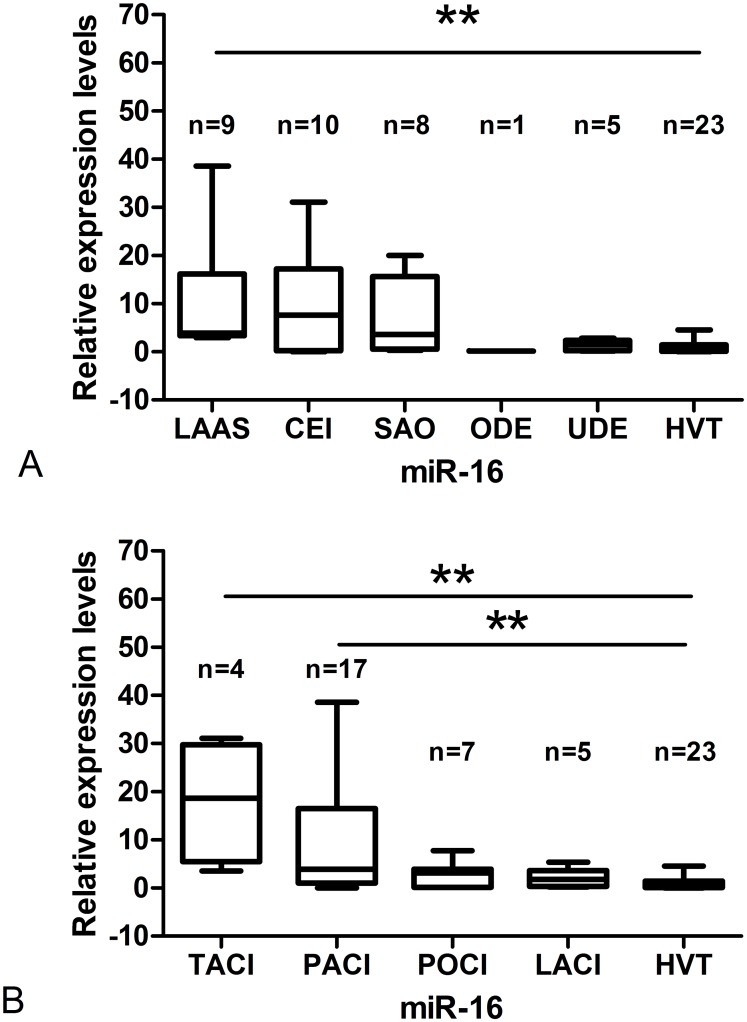
Comparison of miR-16 in HACI patients of different TOAST (OCSP) subtypes with the HVT group. (A)The relative expression levels in different subtypes are shown with minimums, the 25th percentiles, medians, the 75th percentiles, and maximums. The significance level (α) was adjusted to 0.01(0.05/5) in this subpart according to the Mann-Whitney test. (B) The significance level (α) was adjusted to 0.0125(0.05/4) in this subpart according to the Mann-Whitney test.

The patients with HACI were divided into five and four subtypes according to TOAST criteria and OCSP criteria, respectively. The differences between HACI patients in different subtypes and HVT were compared (Fig 7). The expression level of miR-16 in HACI patients of the LAAS subtype was significantly higher than in HVT patients. The expression of miR-16 in LAAS patients was used to draw a ROC curve (Fig 6B) and the AUC was found to be 0.952 (95% CI 0.879–1.024). When the relative expression level of miR-16 was at the optimum truncation point (2.804), sensitivity and specificity were 100% and 91.3%, respectively. MiR-16 expression in the TACI and PACI subtypes of HACI was significantly higher than in HVT. A ROC curve was drawn with data on the expression of plasma miR-16 between HACI patients with TACI/PACI and the HVT group (Fig 6C and 6D). TACI: P = 0.003, AUC = 0.978, (95% CI 0.925–1.031); optimum truncation point = 3.093, sensitivity = 100%, specificity = 91.3%. PACI: P = 0.002, AUC = 0.795, (95% CI 0.642–0.948); optimum truncation point = 2.168, sensitivity = 70.6%, specificity = 87%.

### Mir-16 was associated with stroke prognosis

**Fig 8:**
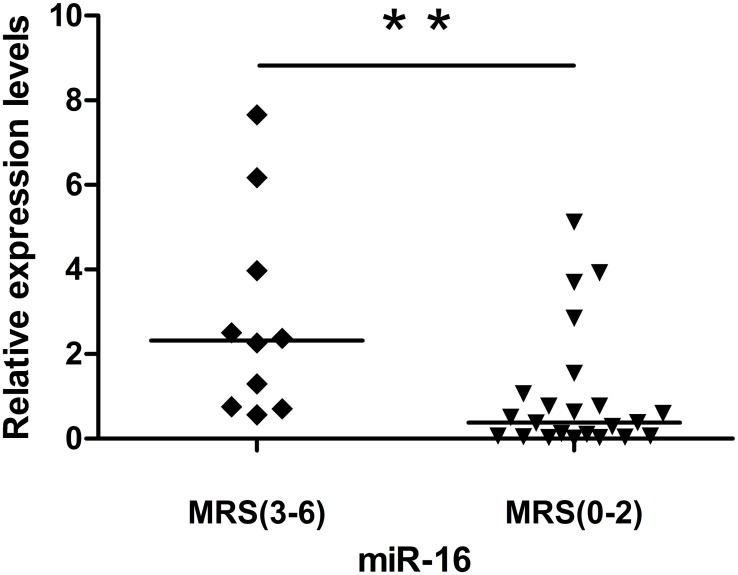
Comparison of miR-16 in HACI patients with good prognoses versus the group with poor prognoses. MiR-16 of the group with poor prognoses was significantly higher than in the group with good prognoses. The straight line in the figure represents the median.

The 33 HACI patients were divided into groups with good prognosis (mRS 0–2, n = 23) and poor prognosis (mRS 3–6, n = 10). The relationship between miR-16 and the prognosis of HACI was studied further (Fig 8). MiR-16 expression was significantly higher in the poor prognosis group than in the good prognosis group.

## Discussion

The treatment and rehabilitation of cerebral infarction has become a serious social burden. Early diagnosis in the hyperacute stage would greatly affect the treatment and management of patients. Our study looked for circulating biomarkers for HACI, and gathered information for new cerebral infarction treatment targets.

Many mature miRNAs circulate in blood plasma [16]. Recently, studies have been published about the search for blood biomarkers of cerebral infarction among miRNAs that are specific to cerebral tissue. In our study, we used microarray technology to compare circulating miRNAs between patients with cerebral infarction and healthy control patients. We found that a portion of the differentially expressed miRNAs in blood after cerebral infarction were specific to cerebral tissue. The microarray results revealed 12 miRNAs with significant differences in expression between HACI and HVT samples. MiR-16 and miR-223 were specific to cerebral tissue and were considered possible candidates as biomarkers of HACI [15]. In a clustering heat map of the differentially expressed miRNAs, four cases of HVT clustered together and could be considered as one group. This illustrated that the specimens of our study were representative. There were 11 significantly up-regulated miRNAs and one significantly down-regulated miRNA. This phenomenon was consistent with the hypothesis that the increased nucleic acids in plasma were derived from cell apoptosis or necrosis [17].

Seven of the 12 miRNAs that showed significant differences in the microarray analysis were chosen for testing by qPCR, because their mean of the normalized values was greater than 1.0 in at least one group. The median, calculated by Ct values, of the seven miRNAs were all higher in the HACI group than in the HVT group, consistent with the general trend of the microarray data, but only miR-16 was validated as having a significant difference between HACI and HVT. There were four clinical features with significant differences between HACI and HVT, but only atrial fibrillation and miR-16 were entered into the final regression model. Atrial fibrillation had no statistical significance in the model. This suggested that the baseline setting of this study was reasonable. It eliminated the possible error caused by the risk factors of cerebral infarction, which have uneven distribution between the two groups. A ROC curve was used to judge the diagnostic accuracy of miR-16 for HACI. Its AUC was 0.775. When the relative expression level of miR-16 was at its optimum truncation point, the sensitivity and the specificity were 69.7% and 87%. The sensitivity and specificity of miR-16 reached 100% and 91.3% in patients with large artery atherosclerosis. In previous studies of the change of circulating blood miRNAs after cerebral infarction, AUC values ranged from 0.642 to 0.999, sensitivity ranged from 80% to 88.3%, and specificity ranged from 41.1% to 94% [18–20]. The sensitivity of this study was relatively high, but the samples included in this study were all taken from HACI patients within 6 hours of infarction onset. The previous three studies included patients within 3 days, 1 day, or 1 day of infarction onset.

GO analysis was applied to gain insight into the biological processes of the target genes of miR-16. Some GO categories affected by miR-16 have been confirmed to be involved in the pathogenesis of acute cerebral infarction. For example, a study [21] showed that programmed cell death 5 (PDCD5) protein was a key regulator of autophagy. It might play an important role after middle cerebral artery occlusion (MCAO) injury. Cheyuo et al. [22] demonstrated that milk fat globule-EGF VIII (MFG-E8) promoted neural stem cell proliferation and migration by adjusting αβ3-integrin following stroke. Another study [23] showed that tricyclodecan-9–yl—xanthogenate (D609) was beneficial after cerebral infarction by inhibiting sphingomyelin synthase (SMS), increasing ceramide levels, and induction of cell-cycle arrest by up-regulating p21 and causing hypophosphorylation of retinoblastoma (Rb). To further explore the broader relationships between miR-16 and the pathogenesis of HACI, pathway analysis was performed. Classical signaling pathways have been reported to be activated following cerebral infarction, for example p53-mediated neuronal apoptosis in post-ischemic brain damage [24]. P53 influenced the binding of NF-κB to p300 and blocked NF-κB-mediated survival signaling. P53 also transferred to mitochondria and caused the release of cytochrome C. Candesartan and glycyrrhizin [25] were proved to alleviate the Toll-like receptor (TLR) pathway after MCAO, including upregulation of TLR-2, TLR-4, Myd88, TRIF, IRF-3, and downregulation of TNF-α, IL-1β, IL-6, and NF-κB. A study [26] proposed that erbB receptors were upregulated in neurons, macrophages, and microglia after cerebral infarction, which might be related to neuroprotection and repair.

MiR-16 expression was significantly higher in HACI patients of the LAAS subtype, compared to HVT. The same was true of HACI patients of the TACI and PACI subtypes. MiR-16 expression was also significantly higher in the group of patients with poor prognoses than in the group with good prognoses. The study of Zeng et al. [18] showed that the expression of miR-210 in human peripheral blood leukocytes was not related to the TOAST criteria and OCSP criteria of acute cerebral infarction (ACI). It was, however, related to the prognosis of ACI. The study of Wang et al. [27] showed that miR-223 expression in peripheral blood leukocytes from cerebral infarction patients of the LAAS and SAO subtypes was significantly higher than in the control group. Further research on the relationship between miRNAs in peripheral blood and peripheral blood cells after cerebral infarction is needed. Hua et al. [15] reported that miR-16 had particularly high expression in the brain’s olfactory bulb, which may relate to our result that miR-16 mainly changed in the anterior circulation after cerebral infarction.

Some researchers have selected endogenous miR-16 as a housekeeping gene for qPCR studies, such as Tsai [28], but there have also been studies [15] [29] that showed particularly high expression of miR-16 in cerebral tissue. The results of our study also clearly show that miR-16 in plasma will significantly increase after cerebral infarction. In such a situation, selecting endogenous miR-16 as a housekeeping gene would add a new systemic error, since expression varies between patients and the control group. Exogenous synthetic cel-miR-54 was selected as a housekeeping gene in our study. It should, in theory, maintain a steady concentration between the patient group and the control group.

A limitation of our study was the small sample size. In addition, we have not explored how specific miR-16 is to cerebral tissue. Finally, the present study did not explore the mechanism in a cell or animal model to validate the predicted target genes of miR-16.

## Conclusions

In conclusion, the present study provided the first clinical evidence, to our knowledge, of circulating miR-16 as a biomarker of HACI. MiR-16 could be used to diagnose HACI with high sensitivity and specificity. Bioinformatic analysis demonstrated that target genes regulated by miR-16 were involved in several biological processes and signaling pathways after HACI. The study offered intriguing new perspectives on the involvement of miRNAs in HACI, but the precise mechanisms need further experimental validation. Plasma concentrations of miR-16 were related to TOAST criteria, OCSP criteria, and the prognosis of HACI patients.

## Abbreviations

AUC: area under curve
CEI: cardioembolic infarct
Ct: cycle threshold
GO: gene ontology
HACI: hyperacute cerebral infarction
HVT: healthy volunteers
KEGG: Kyoto Encyclopedia of Genes and Genomes
LAAS: large artery atherosclerosis
LACI: lacunar cerebral infarct
miRNAs: MicroRNAs
mRS: modified Rankin Score
OCSP: Oxford Community Stroke Project
ODE: other determined etiologies
PACI: partial anterior circulation infarction
POCI: posterior circulation infarct
qRT-PCR: quantitative reverse transcription polymerase chain reaction
ROC: receiver operating characteristic
SAO: small artery occlusion
TACI: total anterior circulation infarction
TLR: Toll-like receptor
TOAST: Trial of org10172 in Acute Stroke Treatment
UDE: undetermined etiology
